# Evaluation of Nutri-Score and NewTools-score in a Norwegian setting using a reference standard based on nutrition experts’ ranking of foods’ healthiness

**DOI:** 10.29219/fnr.v69.11444

**Published:** 2025-03-24

**Authors:** Mari Mohn Paulsen, Lisa Bucher Holm, Anna Amberntsson, Marianne Hope Abel, Lene Frost Andersen

**Affiliations:** 1 Department of Food Safety, Norwegian Institute of Public Health, Oslo, Norway; 2 Centre for Sustainable Diets, Norwegian Institute of Public Health, Oslo, Norway; 3 Department of Nutrition, Institute of Basic Medical Sciences, University of Oslo, Norway; 4 Centre for Evaluation of Public Health Measures, Norwegian Institute of Public Health, Oslo, Norway; 5 Department of Physical Health and Ageing, Norwegian Institute of Public Health, Oslo, Norway

**Keywords:** nutrient profiling, front-of-pack nutrition label, convergent validity, validation, healthiness ratings, food-based dietary guidelines

## Abstract

**Background:**

The Nutri-Score is a color-coded front-of-pack nutrition label that classifies foods and beverages from A (higher nutritional quality) to E (lower nutritional quality). The NewTools-score is an adaptation of the Nutri-Score 2023-version, modified to better align with the Nordic Nutrition Recommendations. Validating nutrient profiling models in different countries is crucial for their reliability and effectiveness in promoting healthier food choices and combating non-communicable diseases.

**Objective:**

This study aimed to assess the convergent validity of the Nutri-Score and the NewTools-score by evaluating their agreement with a reference standard based on rankings of foods’ healthiness by Norwegian nutrition experts. Additionally, we examined the consistency among these experts in rating foods’ healthiness representative of the Norwegian diet.

**Design:**

Between August and December 2023, 29 nutrition experts completed a web-based questionnaire, scoring 100 foods on a scale from 1 (less healthy) to 6 (very healthy) based on the Norwegian food-based dietary guidelines. Agreement among experts was evaluated using descriptive statistics and Cronbach’s alpha. We calculated both Nutri-Score and NewTools-score for all 100 foods and assessed their agreement with the reference standard through cross-classification and score distribution analyses.

**Results:**

The nutrition experts exhibited high agreement in their healthiness ratings of foods representative of the Norwegian diet. The Nutri-Score 2023-version showed good agreement with the experts for most foods, although discrepancies were observed for wholegrain and refined grains, fat content in dairy products, certain fish products, and plant-based dairy and meat substitutes. The NewTools-score displayed overall better agreement with the reference standard for several foods and with fewer discrepancies.

**Conclusions:**

Norwegian nutrition experts showed high agreement in rating the healthiness of foods representative of the Norwegian diet. While the Nutri-Score 2023-version aligned well with experts’ ratings, the NewTools-score demonstrated better agreement than Nutri-Score in this Norwegian context, despite some remaining discrepancies.

## Popular scientific summary

- Nutri-Score is a nutrition label grading foods by nutritional quality, while the NewTools-score adapts Nutri-Score to better align with the Nordic Nutrition Recommendations.
- This study assessed the validity of both scores using a reference standard of food healthiness from nutrition experts.
- Nutri-Score showed good agreement, but the NewTools-score demonstrated even higher agreement with the reference standard.
- This study provides knowledge about the validity of these nutrient profile models, relevant for promoting healthier food choices.

Unhealthy diets are major contributors to the development of non-communicable diseases (NCDs) and significantly contribute to the global burden of disease, posing a substantial risk to both morbidity and mortality (1). Nutrient profiling (NP) is defined as the science of classifying foods according to their nutritional composition for the purpose of promoting health and preventing disease (2). It has several applications, including front-of-pack nutrition labeling (FOPNL) and product reformulation (3).

The World Health Organization endorses FOPNL on foods and beverages as a mean to assist consumers in making more informed nutritional choices by ranking products according to their healthiness (4, 5). Encouraging consumer shifting from less nutritious foods toward healthier options has the potential to not only improve dietary quality but also to reduce the risks associated with NCDs. Moreover, FOPNLs can incentivize manufacturers to enhance the nutritional profiles of their products (4). The Nutri-Score is a summary graded, color-coded FOPNL that classifies foods and beverages from A (higher nutritional quality) to E (lower nutritional quality) (6). Nutri-Score is adopted by public health authorities as a voluntary label in several European countries and is a candidate for a harmonized mandatory FOPNL in Europe (7). Nutri-Score was launched in 2017 (hereafter referred to as Nutri-Score 2017-version) (8), and an updated score was launched in 2023 (hereafter referred to as Nutri-Score 2023-version) (9, 10).

Validating NP models in different countries is crucial for their reliability and effectiveness in promoting healthier food choices and combating NCDs. However, this task presents significant challenges due to the absence of a universally accepted ‘gold standard’ that defines healthy food (2). There are several factors that affect the validity of NP models, including content validity, predictive validity, convergent validity, and face validity (2, 11, 12). The method most used is to examine different foods to assess whether the NP model classifies them appropriately, as a form of content validity (13). This is a less robust method than convergent validity, which involves a comparison of the NP model against other measures, such as national food-based dietary guidelines (FBDGs) or other NP models (13).

Both Nutri-Score 2017 and 2023-versions have been found to identify healthier options within food groups and to have a high discriminating ability across food categories (14, 15). Dréano-Trécant et al. (15) found that Nutri-Score 2017-version discriminated foods according to the FBDGs across eight European countries, including Norway. We previously evaluated the Nutri-Score 2023-version in a Norwegian setting (14) and found that, generally, Nutri-Score’s classification aligned well with the FBDGs. However, some inconsistencies were identified, such as insufficient differentiation between full-fat and reduced-fat cheeses, creams, and processed meats, as well as between whole grain and refined pasta and rice products (14). Moreover, we have gathered insights on the Nutri-Score 2023-version’s performance from various food system actors in Norway, including stakeholders from food production, industry, research and education, and civil society (16). These actors highlighted several concerns, such as the weighting of specific nutrients, perceived inequities in cross-category comparisons, and misalignments with established Norwegian nutritional guidelines and policies (16). Drawing on these findings (14, 16), the NewTools-score has been developed as part of the NewTools project (17), aiming to create more sustainable and healthy food systems. The NewTools-score is based on the Nutri-Score 2023-version algorithms, with some modifications to better align with the Nordic Nutrition Recommendations (18).

Using a reference standard created by nutrition experts to validate NP models has been suggested as an effective and economical approach to assess convergent validity (12, 19). Scarborough et al. (20) conducted a survey of nutrition professionals in the UK to create a reference standard for the healthiness of foods representative of the British diet and then applied this standard to assess and compare eight different NP models (21). Such a study has not previously been conducted in Norway.

The primary aim of this study was to assess the convergent validity of the Nutri-Score and the NewTools-score by evaluating their agreement with a reference standard. This reference standard was created based on how qualified Norwegian nutrition experts rank the healthiness of foods according to the Norwegian FBDGs. Additionally, we aimed to investigate the extent of agreement among these experts regarding the healthiness of foods representative of the Norwegian diet.

## Methods

### Study design and setting

Between August 2023 and December 2023, we invited 46 nutrition experts in Norway to participate in a survey via e-mail. In total, 29 consented to participate, resulting in a 63% response rate. Our aim was not to gather a large number of participants, but rather to identify and include experts with in-depth knowledge of the FBDGs. These individuals were defined as current or former members of the Norwegian National Council of Nutrition, employees working with nutrition in the Norwegian Directorate of Health, and academics from Norwegian universities with active research and education programs in nutrition. The universities included were the University of Oslo and Oslo Metropolitan University (Eastern Norway), the University of Agder (Southern Norway), the University of Bergen (Western Norway), and The Arctic University of Norway (Northern Norway). The participants were offered a gift card of 500 NOK if completing the study.

### Creation of a reference standard

Data were collected with a web-based questionnaire. The questionnaire included a short description of the study, including information about the purpose of creating a reference standard to validate NPs (e.g. Nutri-Score) in a Norwegian setting. The experts were asked to report background information including sex, age, educational attainment, and field of education. In addition, they were asked to report their knowledge of the Norwegian FBDGs (22), ranging from 1 (no knowledge) to 10 (very high knowledge). They were also asked to report whether they had any conflicts of interest.

After completing the background information, the experts were instructed to score 100 food and beverage items according to their alignment with the Norwegian FBDGs (22) on a scale from 1 (less healthy) to 6 (very healthy). The selection of the scale from 1 to 6 was based on previous work by Scarborough et al. (20, 21). Moreover, the experts were asked to score the foods compared to *all* foods, not just within food groups. The number of foods (*n* = 100) was selected based on a combination of previous studies (20–24), and what was considered feasible for the nutrition experts to score within a time frame of approximately 20–30 min. The selection of the 100 foods was based on the most frequently consumed food groups in the national dietary survey Norkost 3 (25). The selection also included foods where we had previously identified challenges with Nutri-Score according to some components and nutrients, such as cereals with different wholegrain content, and cheeses and meat products with different fat content (14, 18). These foods were included also to specifically evaluate the NewTools-score’s ability to address these known limitations. Additionally, our selection included some composite foods and plant-based alternatives where knowledge is lacking about their classification with Nutri-Score compared to other methods (26, 27). Plant-based meat and dairy alternatives have increased in number and sales both in Norway (28) and in Europe (29). The questionnaire included ingredient lists and nutritional content for most of the foods, except single ingredient fruits, vegetables, and potatoes. Nutritional content was expressed per 100 g of product, included energy (kJ), fat (g), saturated fat (g), total sugar (g), fiber (g), protein (g), and salt (g), and were obtained from the Norwegian Food Composition Table 2021 (30). Only generic food names were used to reduce the probability of errors related to brand-specific preferences, as recommended by Thurecht et al. (31). Ingredient lists were sourced from the most commonly available branded products, obtained from the manufacturers’ official website or online grocery stores.

The web-based questionnaire was pilot tested with five nutrition experts. Based on feedback from the pilot testing, a few adjustments were made to the questionnaire. These included providing ingredient lists to enable a more accurate assessment of nutritional quality and degree of processing, and presenting foods and beverages randomly rather than by food group, with only one item shown at a time. A free-text field was also included at the end of the questionnaire for participants to give feedback if desired. Results from the nutrition experts participating in the pilot were not included in the final analyses.

### Data analysis

Nutri-Score 2023-version (9, 10) and NewTools-score (18) were calculated for all 100 foods and beverages, Supplementary files 1 and 2. The differences between Nutri-Score 2023-version and the NewTools score included seven revisions to the algorithms to improve classification of carbohydrate-rich foods, fish, and fat-rich foods (18). Agreement between nutrition experts’ food ratings was evaluated using descriptive statistics and Cronbach’s alpha analysis, with alpha values of ≥0.8 indicating good agreement and ≥0.9 indicating excellent agreement (32). Expert ratings were excluded from the analyses of agreement if the expert gave identical ratings to all items in a food group, as such uniformity can artificially inflate interrater agreement measures like Cronbach’s alpha, particularly in homogeneous groups. Given the non-normal data distribution, descriptive statistics included median scores, interquartile ranges (IQRs), and min and max values. The level of agreement between the nutrition experts served as an indicator of the robustness of the reference standard for validation purposes.

The evaluation of Nutri-Score and NewTools-score was conducted by assessing their level of agreement with the reference standard. This involved cross classification between the experts’ scores and each Nutri-Score and NewTools-score, as well as analyzing the distribution of experts’ scores for foods and beverages within each Nutri-Score and NewTools-score class. Good agreement between the nutrient profiles and the reference standard was defined as:

**Table:** 

Nutri-Score/NewTools-score^1^	Nutrition expert median score
A	6
B	5
C	3 or 4
D	2
E	1

1 Nutri-Score with proposed revisions from the NewTools-project.

Small and large disagreements between the nutrient profiles and the reference standard were defined as:

**Table:** 

Nutri-Score/ NewTools-score^1^	Nutrition expert median score	
	Small disagreement	Large disagreement
A	5	≤ 4
B	4 or 6	≤ 3
C	2 or 5	1 or 6
D	1 or 3	≥ 4
E	2	≥ 3

1 Nutri-Score with proposed revisions from the NewTools-project.

Statistical analyses were conducted in Stata/SE 17.0.

### Ethics

This study was acknowledged by the Norwegian Agency for Shared Services in Education and Research, reference number: 766589. Informed consent was collected from all participants by e-mail. Data on sex were omitted from the analysis due to the disproportionate ratio of women to men to prevent identification of any participants.

## Results

### Participants

Table 1 shows characteristics of the study participants. Most of the participants were between 31 and 60 years old, and all held either a PhD or a master’s degree. All the experts reported having high knowledge about the FBDGs, with the majority (62%) reporting the highest possible level (score 10).

**Table 1:** Characteristics of study participants

Characteristics	*n* (%)
**Age, years**	
18–30	1 (3)
31–45	10 (35)
46–60	14 (48)
> 60	4 (14)
**Educational level**	
Bachelor’s (BA/BSc)	0 (0)
Master’s (MA/MSc)	7 (24)
PhD (Doctorate)	22 (76)
**Field of education**	
Nutrition	28 (97)
Medicine	1 (3)
Other	0 (0)
**Knowledge of FBDGs^1^**	
0–6	0 (0)
7–8	5 (17)
9–10	24 (83)

1 FBDGs: Food-based dietary guidelines. Knowledge of the Norwegian FDBGs was self-reported on a scale from 1 (no knowledge) to 10 (high knowledge).

### Nutrition experts’ scoring of foods

Table 2 presents the nutrition experts’ scores across food categories. The categories ‘vegetables, legumes’, and ‘fish, fish products’ achieved the highest median scores, whereas ‘sweets’ and ‘cakes’ received the lowest median scores. The Cronbach’s alpha coefficient for all foods and beverages combined was 0.98, indicating strong internal consistency among the experts. Across individual food groups, Cronbach’s alpha coefficients were excellent, except for ‘sweets’, where it was considered good (α = 0.86) (32). The food groups ‘fruit, berries, nuts, jam’, ‘bread, bread products’, and ‘potato, potato products’ exhibited the highest alpha coefficients. The full product list, including nutrition experts’ scores for all 100 foods and beverages, along with the median, IQR, mean, and min-max values, is provided in Supplementary file 3.

**Table 2:** Nutrition experts’ scoring within food categories, displaying median and mean scores ranging from 1 (less healthy) to 6 (very healthy), along with measures of internal consistency (Cronbach’s alpha).

Food group	Number of foods	Median (IQR)^1^	Mean	Cronbach’s α
Vegetables, legumes	6	6 (0–2)	5.1	0.98
Fish, fish products	6	5 (0–1)	4.9	0.98
Fruit, berries, nuts, jam	8	4 (0–2)	4.2	0.99
Cheese	6	3 (1–2)	3.7	0.97
Bread, bread products	8	3.5 (1–2)	3.8	0.99
Dairy, dairy alternatives	10	3 (1–2)	3.5	0.98
Oil, butter, margarine	6	3 (0–2)	3.1	0.98
Composite foods, other	11	3 (0–1)	2.7	0.98
Potato, potato products	4	2.5 (1–2)	3.3	0.99
Meat, meat products	9	3 (0–1)	3.1	0.97
Cereals, pasta, rice, flour	9	3 (0–2)	3.4	0.99
Beverages	7	2 (0–1)	2.2	0.99
Sweets	6	1 (0–2)	1.4	0.86
Cakes	4	1 (0–1)	1.2	0.95

1 Median within food group refers to the group median score based on the median scores of all foods in the group.

Foods and beverages with the lowest variability in expert scores (IQR = 0) generally received the highest (median score of 6) and lowest (median score of 1) ratings, such as broccoli and apples (high) and fruit jelly sweets and chocolate muffins (low) (Supplementary file 3). Foods with moderate variability (IQR = 2) generally had intermediate median scores of 3 or 4, including canned corn, peanuts, mayonnaise, and oat drink (Supplementary file 3). Foods and beverages with the highest variability in scores, indicated by wide min-max ranges, were primarily rated low, with median scores of ≤ 3, such as minced red meat, raisins, full-fat dairy products, and butter. Notably, 40% of ‘dairy, dairy alternatives’ exhibited min-max ranges of 4 or more (Supplementary file 3).

### Evaluating agreement of Nutri-Score and NewTools-score with the reference standard

Table 3 presents the agreement between the Nutri-Score 2023-version and the reference standard, using a cross-classification of all scores assigned by the nutrition experts (*n* = 2,900 scores) within each Nutri-Score. Half of the ratings for foods within Nutri-Score A had an expert score of 6, with only 2% receiving a score of 1. Foods within Nutri-Score B most frequently received expert scores of 3 (25%), followed by 4 and 5. In Nutri-Score C, most of the foods and beverages were rated as 2 (30%) or 3 (27%) by the experts. Both Nutri-Score D and E predominantly received expert ratings of 1 (30 and 53%, respectively).

**Table 3:** Cross-classification of 100 foods and beverages by Nutri-Score 2023-version and 29 nutrition experts’ scores (*n* = 2,900 ratings), presented as counts and percentages (*n* (%)).

Nutri-Score	Expert score from very healthy (6) to less healthy (1)						Total (%)
	6 (%)	5 (%)	4 (%)	3 (%)	2 (%)	1 (%)	
A	**333 (50)**	152 (23)	89 (13)	48 (7)	33 (5)	12 (2)	667 (23)
B	56 (11)	94 (19)	115 (23)	**125 (25)**	75 (15)	28 (6)	493 (17)
C	25 (3)	69 (9)	151 (19)	216 (27)	**240 (30)**	111 (14)	812 (28)
D	10 (2)	34 (6)	86 (16)	113 (21)	144 (26)	**164 (30)**	551 (19)
E	3 (1)	22 (6)	25 (7)	32 (8)	95 (25)	**200 (53)**	377 (13)
Total	427 (15)	371 (13)	466 (16)	534 (18)	587 (20)	515 (18)	2,900 (100)

Bold numbers represent the most frequent nutrition expert score within each Nutri-Score.

Table 4 presents the agreement between the NewTools-score and the reference standard, using a similar cross-classification of nutrition experts’ scores within each NewTools-score. Compared to the Nutri-Score 2023-version, the proportion of foods rated as NewTools-score A with an expert score of 6 increased from 50 to 53%, and those rated as NewTools-score E with an expert score of 1 increased from 53 to 62%. Conversely, the proportion of foods rated as class D with an expert score of 1 decreased from 30 to 17%.

**Table 4:** Cross classification of 100 foods and beverages by the NewTools-Score^1^ and 29 nutrition experts’ scores (*n* = 2,900 ratings), presented as counts and percentages (*n* (%)).

NewTools-Score^1^	Expert score from very healthy (6) to less healthy (1)						Total (%)
	6 (%)	5 (%)	4 (%)	3 (%)	2 (%)	1 (%)	
A	**324 (53)**	138 (23)	82 (13)	38 (6)	18 (3)	9 (1)	609 (21)
B	56 (11)	94 (19)	119 (20)	**122 (25)**	76 (15)	26 (5)	493 (17)
C	34 (4)	84 (11)	150 (19)	**217 (28)**	213 (27)	85 (11)	783 (27)
D	8 (2)	50 (10)	94 (18)	128 (25)	**155 (30)**	87 (17)	522 (18)
E	5 (1)	5 (1)	21 (4)	29 (6)	125 (25)	**308 (62)**	493 (17)
Total	427 (15)	371 (13)	466 (16)	534 (18)	587 (20)	515 (18)	2,900 (100)

1 Nutri-Score with proposed revisions from the NewTools-project.

Bold numbers represent the most frequent nutrition expert score within each NewTools-Score.

Table 5 displays the foods and beverages that were characterized as having good agreement between Nutri-Score 2023-version and the reference standard using the definition as described in the Method section. This included 41 of 100 products (41%). Supplementary file 4 shows all 100 foods with median expert score, Nutri-Score 2023-version and the NewTools-score.

**Table 5:** Foods and beverages with good^*^ agreement between Nutri-Score 2023-version and the reference standard.

Food and beverages	Median expert score, (IQR)	Nutri-Score 2023	Food and beverages	Median expert score, (IQR)	Nutri-Score 2023
**Vegetables, legumes**			**Composite foods, other**		
Broccoli	6 (0)	A	Meatballs with pea purée^2^	3 (1)	C
Tomato	6 (0)	A	Instant brown gravy, as served	3 (1)	C
Chickpeas, canned	6 (1)	A	Muesli bar	1 (1)	E
Tomato, canned	6 (1)	A	Microwave popcorn	2 (1)	D
Pickled cucumber	3 (0)	C	Instant bearnaise sauce, as served	2 (1)	D
**Fish, fish products**			**Potato, potato products**		
Cod, raw	6 (0)	A	Potato, raw	6 (1)	A
Salmon, raw	6 (0)	A	**Meat, meat products**		
**Fruit, berries, nuts, jam**			Cooked ham	4 (1)	C
Apple	6 (0)	A	Lasagna, frozen, ready meal	3 (0)	C
Avocado	6 (0)	A	Minced red meat, 13% fat, raw	3 (1)	C
Banana	6 (0)	A	Sausage, red meat, 18% fat	2 (1)	D
Strawberry jam^1^	3 (2)	C	**Cereals, pasta, rice, flour**		
**Cheese**			Wheat flour, whole grain	6 (1)	A
Spreadable cheese, 7% fat	3 (1)	C	Oat muesli with fruit	4 (2)	C
**Bread, bread products**			**Beverages**		
Crisp bread, rye, 100% whole grain	6 (1)	A	Smoothie, 100% fruit	5 (1)	B
Bread, 76-100% whole grain	6 (1)	A	Orange juice	4 (1)	C
Bread, 51-75% whole grain	5 (1)	B	Energy drink^3^	1 (0)	E
Potato-based tortilla	3 (2)	C	Soft-drink, sugar-sweetened	1 (0)	E
**Dairy, dairy alternatives**			**Sweets**		
Semi-skimmed milk, 1% fat	5 (1)	B	Honey	1 (1)	E
Yoghurt, natural, 3.4% fat	5 (1)	B	Chocolate and nut spread	1 (0)	E
Yoghurt strawberry, 3.1% fat	3 (1)	C	Milk chocolate	1 (0)	E
**Oil, butter, margarine**			**Cakes**		
Olive oil	5 (1)	B	Chocolate biscuit	1 (0)	E
Margarine spreadable, 40% fat	4 (2)	C			
Margarine spreadable, 60% fat	3 (2)	C			
Butter, 82% fat	1 (1)	E			

* Good agreement defined as: A = 6, B = 5, C = 3 or 4, D = 2, and E = 1.

1 80% berries, 25 g sugar,

2 Ready-meal,

3 With taurine, caffeine, and B-vitamins.

We found several discrepancies between the Nutri-Score 2023-version classifications and the reference standard. Table 6 shows food and beverage items with small (35%) and large (22%) disagreements between Nutri-Score and the experts, as defined in the method section.

**Table 6:** Food and beverages with small^*^ and large^*^ disagreements between Nutri-Score 2023-version and the reference standard.

Small disagreement*			Large disagreement*		
Food and beverages	Median expert score, (IQR)	Nutri-Score 2023	Food and beverages	Median expert score, (IQR)	Nutri-Score 2023
**Fish, fish products**			**Vegetables, legumes**		
Mackerel in tomato sauce, 60% fish^1^	5 (1)	C	Corn, canned	4 (2)	A
Fish patties, 60% fish	5 (1)	C	**Fish, fish products**		
Fish gratin, frozen, ready meal	4 (1)	B	Smoked salmon	5 (1)	E
**Fruit, berries, nuts, jam**			**Cheese**		
Peanuts, unsalted	5 (2)	A	Whey cheese, 16% fat	3 (2)	E
Raisins	3 (1)	D	Semi-hard cheese, 16% fat	4 (1)	D
Strawberry jam^2^	2 (1)	C	**Bread, bread products**		
Peanuts salted	2 (1)	C	Crisp bread, rye, with filling	4 (0)	D
**Cheese**			Bread, 26-50% whole grain	3 (1)	B
Cottage cheese, 4.3% fat	5 (1)	A	**Dairy, dairy alternatives**		
Semi-hard cheese, 27% fat	3 (1)	D	Oat drink^3^	4 (2)	D
Whey cheese, 28% fat	2 (2)	E	Soy-based yogurt	3 (1)	B
**Bread, bread products**			**Composite foods, other**		
Wheat-based tortilla	2 (1)	C	Falafel, frozen	4 (1)	A
Bread roll, 0-25% whole grain	2 (0)	C	Plant-based burger, soy-based^4^	4 (1)	A
**Dairy, dairy alternatives**			Potato chips	1 (0)	C
Skimmed milk, 0.1% fat	6 (1)	B	Instant tomato soup, as served	3 (1)	B
Sour cream, 18% fat	3 (2)	D	Plant-based sausage, soy-based^4^	3 (1)	B
Whole milk, 3.5% fat	2 (1)	C	**Potato, potato products**		
Sour cream, 35% fat	2 (1)	D	Pommes frites^4^	2 (2)	B
Cream, 37% fat	1 (1)	D	Instant mashed potato, as serve	3 (2)	B
**Oil, butter, margarine**			Meat, meat products		
Mayonnaise	3 (2)	D	Minced meat, chicken, raw	4 (1)	A
Butter-margarine blend, 82% fat	2 (0)	E	**Cereals, pasta, rice, flour**		
**Composite foods, other**			Pasta, refined	2 (1)	A
Pizza, ready meal^4^	2 (1)	C	Wheat flour, refined	2 (1)	A
**Potato, potato products**			Rice, refined	2 (1)	B
Potatoes au gratin	2 (1)	C	Breakfast cereals, oat rings	3 (2)	B
**Meat, meat products**			**Beverages**		
Minced red meat, 5% fat, raw	4 (1)	B	Caffe mocca^6^	2 (1)	D
Liver paté, oven-baked	3 (1)	D	**Sweets**		
Sausage, red meat, 10% fat	3 (1)	D	Ice lolly^7^	1 (1)	C
Meatballs	3 (1)	D			
**Cereals, pasta, rice, flour**					
Rice, brown	5 (1)	A			
Pasta, whole grain	5 (0)	A			
Breakfast cereals, chocolate	2 (1)	C			
**Beverages**					
Soft drink, sugar-free	2 (1)	C			
Fruit squash, as served	2 (1)	E			
**Sweets**					
Pastilles, sugar-free	2 (2)	C			
Fruit jelly sweets	1 (0)	D			
**Cakes**					
Sweet bun, with raisins	1 (1)	D			
Soft flatbread^5^	1 (0)	D			
Chocolate muffin	1 (0)	D			

* Small disagreement is defined as: A = 5, B = 4 or 6, C = 2 or 5, D = 1 or 3, and E = 2. Large disagreement is defined as: A ≤ 4, B ≤ 3, C = 1 or 6, D ≥ 4, and E ≥ 3.

1 Canned,

2 54% berries, 29 g sugar,

3 With calcium, vitamin D, B2, and B12,

4 Frozen,

5 With sugar and cinnamon,

6 1.1% fat, 9% sugar,

7 With chocolate coating.

In the ‘vegetables, legumes’ category, good agreement was seen for all products except canned corn, which was characterized as a large disagreement. For the cheeses, only one product was characterized as good agreement, whereas the other had small or large disagreements. Nutri-Score 2023-version discriminated neither between the full-fat and fat-reduced semi-hard cheeses included in the survey (class D) nor whey cheeses (class E). However, the nutrition experts discriminated between these cheeses, giving a median score of 4 for fat-reduced and 3 for full-fat semi-hard cheese, and 2 and 1 for fat-reduced and full-fat whey cheese, respectively. Nutri-Score 2023-version for fish and fish products had several deviations from the reference standard. Large disagreements within the ‘bread, bread products’ category were seen for bread with 26–50% whole grain and crisp bread with rye filling. Within the ‘dairy, dairy alternatives’ category, large disagreements were seen for some plant-based alternatives. Nutri-Score 2023-version did not discriminate between full-fat and fat-reduced sour cream (class D), or between milk with 1 and 0.1% fat content (class B), whereas the experts scored the sour creams with 2 and 3, and the milks with median scores of 5 and 6, respectively. The ‘composite foods, other’ was the food category that had most disagreements between Nutri-Score and the experts. This was particularly seen for some plant-based products that received Nutri-Score A but a median expert score 4. The ‘potato, potato products’ category showed good agreement for raw potatoes (A and 6), but large disagreements for pommes frites (B and 2) and instant mashed potatoes (B and 3). For ‘cereals, pasta, rice, flour’, Nutri-Score classified all products of wheat flour and pasta as A regardless of whole grain content, whereas the experts differed by scoring the whole grain pasta and flour as 5 and 6, respectively. Rice also showed discrepancies in the ratings with brown rice obtaining A and median expert score 5, whereas refined rice obtained class B and median expert score 2.

In total, 86% of the 100 food and beverage items received the same score from both the Nutri-Score 2023-version and the NewTools-score. Table 7 displays median expert scores for foods and beverages that received a different NewTools-score compared to the Nutri-Score 2023-version. Overall, the NewTools-Score seemed to have better agreement with the reference standard than the Nutri-Score 2023-version. Several foods and beverages that were given a low median expert rating obtained a poorer NewTools-score compared to the Nutri-Score 2023-version (Table 7).

**Table 7:** Median expert score for food and beverage items that change score with the NewTools-Score^1^ compared to Nutri-Score 2023-version (*n* = 14).

Food and beverages	Median expert score (IQR)	Nutri-Score 2023	NewTools-score^1^
Smoked salmon	5 (1)	E	D
Rice, brown	5 (1)	A	C
Mayonnaise	3 (2)	D	C
Instant tomato soup, as served	3 (1)	B	C
Strawberry jam, light, 54% berries, 29 g sugar	2 (1)	C	D
Sour cream, 35% fat	2 (1)	D	E
Rice, refined	2 (1)	B	C
Pasta, refined	2 (1)	A	B
Breakfast cereals, chocolate	2 (1)	C	D
Cream, 37% fat	1 (1)	D	E
Ice lolly with chocolate coating	1 (1)	C	D
Fruit jelly sweets	1 (0)	D	E
Soft flatbread, with sugar and cinnamon	1 (0)	D	E
Chocolate muffin	1 (0)	D	E

1 Nutri-Score with proposed revisions from the NewTools-project.

The NewTools-score differentiated between refined and whole grain pasta more accurately according to nutrition experts’ ratings: refined pasta obtained class B, while whole grain pasta retained class A. In contrast, both types of pasta received class A in the Nutri-Score 2023-version (Supplementary file 4). However, both refined and whole grain wheat flour obtained class A with both the Nutri-Score 2023-version and the NewTools-score, not reflecting the differences in healthiness as rated by experts (Supplementary file 4). For rice, the Nutri-Score 2023-version distinguished between refined and whole grain rice, assigning class B to refined rice and class A to whole grain rice. However, the NewTools-score assigned both refined and whole grain rice to class C, removing the distinction made by Nutri-Score and failing to reflect the experts’ differentiation (median scores of 2 and 5, respectively).

Sour cream and full-fat cream both obtained lower NewTools-scores compared to the Nutri-Score 2023-version, allowing for discrimination between full-fat and fat-reduced versions of sour cream as reflected in the reference standard. However, semi-hard cheeses received class D with both scoring systems regardless of fat-content, failing to differentiate between these products according to the reference standard.

## Discussion

This study demonstrated that qualified Norwegian nutrition experts generally had a high level of agreement in their scoring of the healthiness of foods representative of the Norwegian population’s diet. Agreement between Nutri-Score 2023-version and the ranking by the nutrition experts was high for most foods and beverages. However, some discrepancies were observed, including for wholegrain versus refined versions of flour and pasta, full-fat and reduced-fat versions of cheeses and creams, certain fish products, and plant-based meat and dairy substitutes. The NewTools-score showed higher agreement with the reference standard for several foods.

### Agreement between the nutrition experts

The present study constructed a reference standard to serve as a tool for evaluating NP models. Although the World Health Organization has suggested that using a reference standard based on nutrition experts’ ranking is an effective and economical approach to validating NP models (15, 16), there is no consensus or guidelines on how to construct such a standard, and different methodologies have been used (1–5). Achieving a high level of consensus among the nutrition experts in the present study was considered important to support the credibility of the use as a reference standard. If expert agreement had been low, the differences observed among the Nutri-Score 2023-version, the NewTools-score, and the reference standard would not necessarily have indicated poor validity in the NP models’ ability to classify foods correctly according to the FBDGs. It is important to note that the level of expert agreement does not directly confirm the accuracy of the experts’ food ratings in relation to the Norwegian FBDGs but rather reflects the consistency of their ratings, which, in this study, was interpreted as an indication of the reference standard’s robustness.

We found the agreement between the experts to be high, with an overall Cronbach’s alpha of 0.98, and Cronbach’s alpha ≥0.84 when analyzing food groups individually. Additionally, the IQR for individual foods was predominantly low (0 or 1), indicating minimal variability between the ratings. Comparable studies that have assessed nutrition experts’ opinions on the healthiness of foods have reported varying expert agreement. Consistent with the findings of Scarborough et al. (20), our study observed that foods with higher variability in nutrition experts’ scores, denoted by IQRs of 2, were primarily those with moderate healthiness ratings, usually reflected by median scores of 3 and 4. Similarly, Scarborough et al. noted that foods assigned mid-range healthiness ratings demonstrated larger standard deviations compared to those rated at the highest and lowest ends of the healthiness spectrum (20).

Notably, despite receiving low median expert scores of 2 and 1, respectively, full-fat dairy products and butter displayed the widest range in expert ratings, spanning from 1 to 6. This indicates that some nutrition experts considered these items to be very healthy, contrary to the explicit recommendations in the Norwegian FBDGs, where low-fat dairy products are recommended over full-fat varieties and soft margarine over butter (22).

### Agreement among Nutri-Score, the NewTools-score, and the nutrition experts

In total, 42% of the foods and beverages had good agreement between Nutri-Score 2023-version and the reference standard, while 22% showed large disagreements. Comparing the NewTools-score to the reference standard, the proportion of foods with good agreement increased to 47%, and those with large disagreements fell to 17%. In this study, we focused on the Norwegian FBDGs, which may differ from those of other countries. These variations could influence how well Nutri-Score aligns with different national guidelines. While Nutri-Score is adopted by several European countries and aims to incorporate aspects of various FBDGs, differences in dietary recommendations may affect its applicability and effectiveness across different nutritional contexts. Briefly, the Norwegian FBDGs focus on recommending an increased consumption of vegetables, fruits and berries, whole grain, and fish, to limit the consumption of processed meat, red meat, salt, and sugar and to choose reduced fat dairy and meat products and soft/fluid fats and oils, and water is recommended for thirst-quenching (22). Previous research has found that both the Nutri-Score 2017- and 2023-versions categorize foods satisfactorily in line with the FBDGs of several countries, including Norway (14, 15, 33, 34). Overall, in the present study, the nutrition experts were slightly more cautious in giving foods the highest scores (5 and 6) compared to Nutri-Score 2023-version and the NewTools-score (class A or B).

The ‘vegetable, legumes’ category had the highest proportion of foods with good agreement, whereas several deviations were observed in the cereals, cheese, and fish categories. The experts rated whole grain and refined versions of flour, pasta, and rice differently, with the whole grain versions receiving median expert scores of 5 or 6 and the refined versions scoring 2. This is consistent with the previous study by Øvrebø et al. (14), which concluded that Nutri-Score 2023-version does not differentiate between whole grain and refined versions of pasta and flour. The revision of the Nutri-Score algorithm for the 2023-version aimed to better distinguish wholegrain from refined grain products, including pasta, rice, flour, and bread (10, 35). The Scientific Committee developing the algorithms for Nutri-Score acknowledged the difficulty in distinguishing some whole grain and refined grain products but prioritized improving the differentiation of bread products (10, 35). The NewTools-score (18) was able to better discriminate between refined and whole grain versions of pasta, compared to the Nutri-Score 2023-version. However, the differentiation between rice products based on whole grain content disappeared with the proposed NewTools-score. The ability to discriminate whole grain from refined products is particularly relevant in a Nordic context, where the Nordic Nutrition Recommendations advocate for consuming at least 90 g whole grain per day due to the well-documented health benefits (36). However, whole grain and refined wheat flours were not differentiated by either the Nutri-Score 2023-version or the NewTools-score, due to the high content of fiber in the refined wheat flour included in this study (5 g/100 g). Analyzing results from two larger food databases in Norway (TradeSolution and Unil), including 425 flour products, showed that the majority of the refined flour products were moved from class A to B or C with the NewTools-score, compared to the Nutri-Score 2023-version (18).

The deviations between both Nutri-Score 2023-version and the NewTools-score and the reference standard seen in the present study for cheeses show that the nutrition experts, in general, rank cheese as being somewhat healthier than the Nutri-Score and the NewTools-score. In the Australian NP model Health Star rating, cheeses are treated with a specific algorithm that shifts the distribution of cheese scores toward better ratings (37). Cheese consumption is high in the Norwegian population and a significant contributor to saturated fat intake (25), making it important to guide consumers toward healthier options within the cheese category.

Several small and large deviations between the Nutri-Score 2023-version and the nutrition experts were observed for fish products. A comparative study across eight European countries (15) found that Nutri-Score 2017-version classified most fish products in classes A and B. However, processed fish products generally receive a lower Nutri-Score class than unprocessed fish products (33, 38). In Norway, increasing fish consumption, including using fish as a bread spread, has been a pronounced public health goal for several years (22, 39). Several smoked salmon products, which, in the current study, received a median expert score of 5 and Nutri-Score E, are marked with the Keyhole label, suggesting that they are recommended products. However, smoked salmon has a very high salt content (1.2–4 g per 100 g), which explains its low Nutri-Score classification. With salt consumption in Norway exceeding recommended intake (25), this raises the question of whether it is beneficial to encourage the consumption of high-salt fish products. With the NewTools-score, smoked salmon will obtain a score D or C (depending on the salt content), rather than E, due to a reward for its fish content (18).

In the present study, plant-based dairy and meat substitutes also showed several deviations between the experts and both the Nutri-Score 2023-version and the NewTools-score, where the experts mostly rated the products as less healthy. Huybers et al. (27) found that the Nutri-Score 2023-version better aligned with the Dutch FBDGs regarding plant-based options compared to the Nutri-Score 2017-version, although challenges remained concerning often low levels of micronutrients, high salt, and low protein content in products obtaining av favorable Nutri-Score. A Swedish study revealed significant discrepancies between how the Nordic Keyhole and the Nutri-Score 2023-version classified plant-based meat and fish substitutes, with 67% of products that were ineligible for the Keyhole receiving a Nutri-Score of A or B (26). Given the increased awareness of more sustainable diets, a diet richer in plant-based foods and lower in animal-based foods has gained popularity (40). This makes accurate categorization of these foods and beverages important.

Several other foods and beverages in the present study also changed class when using the NewTools-score compared to the Nutri-Score 2023-version. These included full-fat creams (from D to E), strawberry jam (from C to E), mayonnaise (from D to C), and several typical unhealthy products, such as breakfast cereals with chocolate and ice lollies (from C to D), and fruit jelly sweets, sugar and cinnamon flat bread, and chocolate muffins (from D to E). These changes improved the agreement with the nutrition experts. The inability of Nutri-Score 2023-version to discriminate between regular and fat-reduced creams has previously been identified in a Norwegian context (14, 16). Additionally, actors in the Norwegian food system have raised concerns about the high sugar content allowed by the Nutri-Score algorithm, resulting in some products relatively high in sugar still obtaining a good score (16).

All in all, the Nutri-Score 2023-version showed a good agreement with the experts’ ranking, which can be considered as crucial to obtain trust in the label if it is to be implemented. Moreover, the NewTools-score improved the agreement and removed some of the larger discrepancies in the ranking. However, several discrepancies remain unresolved.

## Strengths and limitations

Strengths of the current study include the involvement of specifically selected nutrition experts working in health authorities, as well as employees at universities conducting research and higher education in nutrition sciences who have in-depth knowledge of the FBDGs. The inclusion of food items was based on the latest national dietary survey, Norkost 3 (7), and reflects a representative selection of foods and beverages consumed by the Norwegian population. The relatively large range of included food and beverage items made it possible to investigate specific categories such as full-fat and reduced-fat cheeses, smoked salmon, and plant-based substitutes, offering insights into where Nutri-Score 2023-version and the NewTools-score work well and where they have limitations.

A limitation of the study is the lack of a gold standard for testing agreement between NP models and a reference standard based on nutrition experts. Consequently, the definitions of good agreement, and small and large disagreements were based on somewhat subjective criteria. Furthermore, the focus on the Norwegian population may limit the generalizability of the results to other contexts with different food cultures and dietary guidelines. This study is primarily suited for evaluating whether food categories are logically positioned on the Nutri-Score scale and may not be as well-suited for assessing how effectively the Nutri-Score distinguishes between products within a category. However, we included certain food items that were specifically selected based on previously observed challenges in differentiating Nutri-Score ratings within categories (e.g. full-fat vs. reduced-fat cheese) (14, 16). As a result, we were still able to test, to some extent, how well the Nutri-Score performs within categories. This study included only 100 food items, distributed across 14 food categories, with each category containing 4 to 11 items. The limited number of food items could have influenced the results, and the agreement within food groups might have varied if different or additional food items were included in the questionnaire. The inclusion of foods previously identified as challenging for the Nutri-Score in a Norwegian setting (14, 18) may have influenced the results. This was an intentional choice to specifically test the efficacy of the NewTools-score in addressing known limitations. However, this focus could limit the generalizability of the findings to broader food categories.

## Conclusion

Qualified Norwegian nutrition experts had a high level of agreement in their scoring of the healthiness of foods representative of the Norwegian population’s diet. In a Norwegian setting, agreement among Nutri-Score 2023-version, the NewTools-score, and the nutrition experts was good for most of the food items. However, some discrepancies were observed, particularly for wholegrain and refined versions of flour and pasta, full-fat and fat-reduced versions of cheese and creams, certain fish products, and plant-based meat and dairy substitutes. The NewTools-score improved agreement between the experts and Nutri-Score for several food items, although some discrepancies remain.

## Supplementary Material


