# Supplementary material for: Evaluation of Nutri-Score and NewTools-score in a Norwegian setting using a reference standard based on nutrition experts’ ranking of foods’ healthiness
Source: Food Nutr Res. 2025 Mar 24;69:10.29219/fnr.v69.11444. doi: 10.29219/fnr.v69.11444 (PMC12013598; doi:10.29219/fnr.v69.11444)
Supplement: Supplementary file 1 [file FNR-69-11444-s1.docx]

Supplementary file 3: Nutrition experts’ scores for all 100 foods and beverages, along with the median, IQR, mean, and min-max values

| **Food and beverages** | **Median (IQR)** | **Mean** | **Min,Max** |
| --- | --- | --- | --- |
| **Vegetables, legumes** |  |  |  |
| Broccoli | 6 (0) | 6.0 | 5, 6 |
| Tomato | 6 (0) | 5.9 | 5, 6 |
| Chickpeas, canned | 6 (1) | 5.6 | 4, 6 |
| Tomato, canned | 6 (1) | 5.5 | 4, 6 |
| Corn, canned | 4 (2) | 4.3 | 1, 6 |
| Pickled cucumber | 3 (0) | 3.2 | 1, 5 |
| **Fish, fish products** |  |  |  |
| Cod, raw | 6 (0) | 5.9 | 5, 6 |
| Salmon, raw | 6 (0) | 5.7 | 4, 6 |
| Mackerel in tomato sauce, 60% fish canned | 5 (1) | 5.3 | 4, 6 |
| Fish patties, 60% fish | 5 (1) | 4.6 | 3, 6 |
| Smoked salmon | 5 (1) | 4.4 | 3, 5 |
| Fish gratin, frozen, ready meal | 4 (1) | 3.8 | 2, 6 |
| **Fruit, berries, nuts, jam** |  |  |  |
| Apple | 6 (0) | 5.9 | 5, 6 |
| Avocado | 6 (0) | 5.8 | 4, 6 |
| Banana | 6 (0) | 5.8 | 4, 6 |
| Peanuts, unsalted | 5 (2) | 4.9 | 3, 6 |
| Raisins | 3 (1) | 3.4 | 1, 6 |
| Strawberry jam, 80% berries, 25 g sugar | 3 (2) | 3.1 | 1, 5 |
| Strawberry jam, light, 54% berries, 29 g sugar | 2 (1) | 2.4 | 1, 4 |
| Peanuts salted | 2 (1) | 2.4 | 1, 5 |
| **Cheese** |  |  |  |
| Cottage cheese, 4.3% fat | 5 (1) | 5.1 | 3, 6 |
| Semi-hard cheese, 16% fat | 4 (1) | 4.3 | 3, 6 |
| Semi-hard cheese, 27% fat | 3 (1) | 3.4 | 2, 6 |
| Spreadable cheese, 7% fat | 3 (1) | 3.2 | 1, 5 |
| Whey cheese, 16% fat | 3 (2) | 3.2 | 1, 6 |
| Whey cheese, 28% fat | 2 (2) | 2.7 | 1, 5 |
| **Bread, bread products** |  |  |  |
| Crisp bread, rye, 100% whole grain | 6 (1) | 5.7 | 4, 6 |
| Bread, 75-100% whole grain | 6 (1) | 5.4 | 3, 6 |
| Bread, 50-75% whole grain | 5 (1) | 4.7 | 3, 6 |
| Crisp bread, rye, with filling | 4 (0) | 3.5 | 2, 5 |
| Bread, 25-50% whole grain | 3 (1) | 3.3 | 2, 5 |
| Potato-based tortilla | 3 (2) | 3.3 | 2, 5 |
| Wheat-based tortilla | 2 (1) | 2.5 | 1, 4 |
| Bread roll, 0-25% whole grain | 2 (0) | 1.9 | 1, 3 |
| **Dairy, dairy alternatives** |  |  |  |
| Skimmed milk, 0.1% fat | 6 (1) | 5.5 | 3, 6 |
| Semi-skimmed milk, 1% fat | 5 (1) | 4.9 | 3, 6 |
| Yoghurt, natural, 3.4% fat | 5 (1) | 4.6 | 2, 6 |
| Oat drink with calcium, vitamin D, B2 and B12 | 4 (2) | 3.9 | 2, 6 |
| Soy-based yohurt | 3 (1) | 3.3 | 1, 5 |
| Yoghurt strawberry, 3.1% fat | 3 (1) | 3.2 | 2, 5 |
| Sour cream, 18% fat | 3 (2) | 3.1 | 2, 5 |
| Whole milk, 3.5% fat | 2 (1) | 2.5 | 1, 6 |
| Sour cream, 35% fat | 2 (1) | 1.8 | 1, 6 |
| Cream, 37% fat | 1 (1) | 1.7 | 1, 6 |
| **Oil, butter, margarine** |  |  |  |
| Olive oil | 5 (1) | 5.2 | 3, 6 |
| Margarine spreadable, 40% fat | 4 (2) | 3.7 | 1, 5 |
| Mayonnaise | 3 (2) | 3.2 | 1, 5 |
| Margarine spreadable, 60% fat | 3 (2) | 3.0 | 1, 5 |
| Butter-margarine blend, 82% fat | 2 (0) | 2.1 | 1, 4 |
| Butter, 82% fat | 1 (1) | 1.6 | 1, 6 |
| **Composite foods, other** |  |  |  |
| Falafel, frozen | 4 (1) | 4.3 | 1, 6 |
| Plant-based burger, soy-based, frozen | 4 (1) | 3.6 | 1, 5 |
| Instant tomato soup, as served | 3 (1) | 3.3 | 1, 5 |
| Plant-based sausage, soy-based, frozen | 3 (1) | 3.2 | 1, 5 |
| Meatballs with pea purée, ready-meal | 3 (1) | 3.2 | 2, 5 |
| Instant brown gravy, as served | 3 (1) | 2.7 | 1, 5 |
| Pizza, ready meal, frozen | 2 (1) | 2.3 | 1, 3 |
| Microwave popcorn | 2 (1) | 2.2 | 1, 4 |
| Instant bearnaise sauce, as served | 2 (1) | 1.6 | 1, 3 |
| Muesli bar | 1 (1) | 1.5 | 1, 2 |
| Potato chips | 1 (0) | 1.3 | 1, 4 |
| **Potato, potato products** |  |  |  |
| Potato, raw | 6 (1) | 5.5 | 4, 6 |
| Instant mashed potato, as served | 3 (2) | 2.9 | 1, 5 |
| Potatoes au gratin | 2 (1) | 2.4 | 1, 4 |
| Pommes frites, frozen | 2 (2) | 2.2 | 1, 4 |
| **Meat, meat products** |  |  |  |
| Minced meat, chicken, raw | 4 (1) | 4.5 | 3, 6 |
| Minced red meat, 5% fat, raw | 4 (1) | 3.8 | 2, 6 |
| Cooked ham | 4 (1) | 3.6 | 2, 5 |
| Liver paté, oven-baked | 3 (1) | 3.0 | 1, 5 |
| Lasagna, frozen, ready meal | 3 (0) | 3.0 | 2, 5 |
| Minced red meat, 13% fat, raw | 3 (1) | 2.9 | 1, 6 |
| Sausage, red meat, 10% fat | 3 (1) | 2.8 | 1, 4 |
| Meatballs | 3 (1) | 2.6 | 1, 5 |
| Sausage, red meat, 18% fat | 2 (1) | 1.6 | 1, 3 |
| **Cereals, pasta, rice, flour** |  |  |  |
| Wheat flour, whole grain | 6 (1) | 5.3 | 3, 6 |
| Rice, brown | 5 (1) | 5.0 | 2, 6 |
| Pasta, whole grain | 5 (0) | 4.8 | 2, 6 |
| Oat muesli with fruit | 4 (2) | 3.8 | 2, 6 |
| Breakfast cereals, oat rings | 3 (2) | 2.9 | 1, 5 |
| Pasta, refined | 2 (1) | 2.5 | 1, 4 |
| Wheat flour, refined | 2 (1) | 2.2 | 1, 4 |
| Rice, refined | 2 (1) | 2.2 | 1, 3 |
| Breakfast cereals, chocolate | 2 (1) | 1.7 | 1, 3 |
| **Beverages** |  |  |  |
| Smoothie, 100% fruit | 5 (1) | 4.8 | 2, 6 |
| Orange juice | 4 (1) | 3.8 | 1, 6 |
| Soft drink, sugar-free | 2 (1) | 2.4 | 1, 5 |
| Caffe mocca | 2 (1) | 2.1 | 1, 5 |
| Fruit squash, as served | 2 (1) | 1.7 | 1, 4 |
| Energy drink, with taurin, caffein, and B-vitamins | 1 (0) | 1.1 | 1, 2 |
| Soft-drink, sugar-sweetened | 1 (0) | 1.0 | 1, 1 |
| **Sweets** |  |  |  |
| Pastilles, sugar-free | 2 (2) | 1.97 | 1, 4 |
| Honey | 1 (1) | 1.8 | 1, 6 |
| Ice lolly with chocolate coating | 1 (1) | 1.4 | 1, 3 |
| Chocolate and nut spread | 1 (0) | 1.2 | 1, 3 |
| Milk chocolate | 1 (0) | 1.2 | 1, 3 |
| Fruit jelly sweets | 1 (0) | 1.0 | 1, 2 |
| **Cakes** |  |  |  |
| Sweet bun, with raisins | 1 (1) | 1.5 | 1, 3 |
| Soft flatbread, with sugar and cinnamon | 1 (0) | 1.2 | 1, 2 |
| Chocolate biscuit | 1 (0) | 1.1 | 1, 2 |
| Chocolate muffin | 1 (0) | 1.0 | 1, 2 |

Supplementary file 4: All 100 foods with median expert score, Nutri-Score 2023-version and the NewTools-score^1^

| **Food and beverages** | **Median expert score (IQR)** | **Nutri-Score 2023** | **NewTools- score^1^** |
| --- | --- | --- | --- |
| **Vegetables, legumes** |  |  |  |
| Broccoli | 6 (0) | A | A |
| Tomato | 6 (0) | A | A |
| Chickpeas, canned | 6 (1) | A | A |
| Tomato, canned | 6 (1) | A | A |
| Corn, canned | 4 (2) | A | A |
| Pickled cucumber | 3 (0) | C | C |
| **Fish, fish products** |  |  |  |
| Cod, raw | 6 (0) | A | A |
| Salmon, raw | 6 (0) | A | A |
| Mackerel in tomato sauce, 60% fish canned | 5 (1) | C | C |
| Fish patties, 60% fish | 5 (1) | C | C |
| Smoked salmon | 5 (1) | E | D |
| Fish gratin, frozen, ready meal | 4 (1) | B | B |
| **Fruit, berries, nuts, jam** |  |  |  |
| Apple | 6 (0) | A | A |
| Avocado | 6 (0) | A | A |
| Banana | 6 (0) | A | A |
| Peanuts, unsalted | 5 (2) | A | A |
| Raisins | 3 (1) | D | D |
| Strawberry jam, 80% berries, 25 g sugar | 3 (2) | C | C |
| Strawberry jam, light, 54% berries, 29 g sugar | 2 (1) | C | D |
| Peanuts salted | 2 (1) | C | C |
| **Cheese** |  |  |  |
| Cottage cheese, 4.3% fat | 5 (1) | A | A |
| Semi-hard cheese, 16% fat | 4 (1) | D | D |
| Semi-hard cheese, 27% fat | 3 (1) | D | D |
| Whey cheese, 16% fat | 3 (2) | E | E |
| Whey cheese, 28% fat | 2 (2) | E | E |
| **Bread, bread products** |  |  |  |
| Crisp bread, rye, 100% whole grain | 6 (1) | A | A |
| Bread, 75-100% whole grain | 6 (1) | A | A |
| Bread, 50-75% whole grain | 5 (1) | B | B |
| Crisp bread, rye, with filling | 4 (0) | D | D |
| Bread, 25-50% whole grain | 3 (1) | B | B |
| Potato-based tortilla | 3 (2) | B | B |
| Wheat-based tortilla | 2 (1) | C | C |
| Bread roll, 0-25% whole grain | 2 (0) | C | C |
| **Dairy, dairy alternatives** |  |  |  |
| Skimmed milk, 0.1% fat | 6 (1) | B | B |
| Semi-skimmed milk, 1% fat | 5 (1) | B | B |
| Yoghurt, natural, 3.4% fat | 5 (1) | B | B |
| Oat drink with calcium, vitamin D, B2 and B12 | 4 (2) | D | D |
| Soy-based yohurt | 3 (1) | B | B |
| Yoghurt strawberry, 3.1% fat | 3 (1) | C | C |
| Sour cream, 18% fat | 3 (2) | D | D |
| Whole milk, 3.5% fat | 2 (1) | C | C |
| Sour cream, 35% fat | 2 (1) | D | E |
| Cream, 37% fat | 1 (1) | D | E |
| **Oil, butter, margarine** |  |  |  |
| Olive oil | 5 (1) | B | B |
| Margarine spreadable, 40% fat | 4 (2) | C | C |
| Mayonnaise | 3 (2) | D | C |
| Margarine spreadable, 60% fat | 3 (2) | C | C |
| Butter-margarine blend, 82% fat | 2 (0) | E | E |
| Butter, 82% fat | 1 (1) | E | E |
| **Composite foods, other** |  |  |  |
| Falafel, frozen | 4 (1) | A | A |
| Plant-based burger, soy-based, frozen | 4 (1) | A | A |
| Instant tomato soup, as served | 3 (1) | B | C |
| Plant-based sausage, soy-based, frozen | 3 (1) | B | B |
| Meatballs with pea purée, ready-meal | 3 (1) | C | C |
| Instant brown gravy, as served | 3 (1) | C | C |
| Pizza, ready meal, frozen | 2 (1) | C | C |
| Microwave popcorn | 2 (1) | D | D |
| Instant bearnaise sauce, as served | 2 (1) | D | D |
| Muesli bar | 1 (1) | E | E |
| Potato chips | 1 (0) | C | C |
| **Potato, potato products** |  |  |  |
| Potato, raw | 6 (1) | A | A |
| Instant mashed potato, as served | 3 (2) | B | B |
| Potatoes au gratin | 2 (1) | C | C |
| Pommes frites, frozen | 2 (2) | B | B |
| **Meat, meat products** |  |  |  |
| Minced meat, chicken, raw | 4 (1) | A | A |
| Minced red meat, 5% fat, raw | 4 (1) | B | B |
| Cooked ham | 4 (1) | C | C |
| Liver paté, oven-baked | 3 (1) | D | D |
| Lasagna, frozen, ready meal | 3 (0) | C | C |
| Minced red meat, 13% fat, raw | 3 (1) | C | C |
| Sausage, red meat, 10% fat | 3 (1) | D | D |
| Meatballs | 3 (1) | D | D |
| Sausage, red meat, 18% fat | 2 (1) | D | D |
| **Cereals, pasta, rice, flour** |  |  |  |
| Wheat flour, whole grain | 6 (1) | A | A |
| Rice, brown | 5 (1) | A | C |
| Pasta, whole grain | 5 (0) | A | A |
| Oat muesli with fruit | 4 (2) | C | C |
| Breakfast cereals, oat rings | 3 (2) | B | B |
| Pasta, refined | 2 (1) | A | B |
| Wheat flour, refined | 2 (1) | A | A |
| Rice, refined | 2 (1) | B | C |
| Breakfast cereals, chocolate | 2 (1) | C | D |
| **Beverages** |  |  |  |
| Smoothie, 100% fruit | 5 (1) | B | B |
| Orange juice | 4 (1) | C | C |
| Soft drink, sugar-free | 2 (1) | C | C |
| Caffe mocca | 2 (1) | B | B |
| Fruit squash, as served | 2 (1) | E | E |
| Energy drink, with taurin, caffein, and B-vitamins | 1 (0) | E | E |
| Soft-drink, sugar-sweetened | 1 (0) | E | E |
| **Sweets** |  |  |  |
| Pastilles, sugar-free | 2 (2) | C | C |
| Honey | 1 (1) | E | E |
| Ice lolly with chocolate coating | 1 (1) | C | D |
| Chocolate and nut spread | 1 (0) | E | E |
| Milk chocolate | 1 (0) | E | E |
| Fruit jelly sweets | 1 (0) | D | E |
| **Cakes** |  |  |  |
| Sweet bun, with raisins | 1 (1) | D | D |
| Soft flatbread, with sugar and cinnamon | 1 (0) | D | E |
| Chocolate biscuit | 1 (0) | E | E |
| Chocolate muffin | 1 (0) | D | E |

^1^Nutri-Score with proposed revisions from the NewTools-project
